# Synergism Through WEE1 and CHK1 Inhibition in Acute Lymphoblastic Leukemia

**DOI:** 10.3390/cancers11111654

**Published:** 2019-10-25

**Authors:** Andrea Ghelli Luserna Di Rorà, Matteo Bocconcelli, Anna Ferrari, Carolina Terragna, Samantha Bruno, Enrica Imbrogno, Neil Beeharry, Valentina Robustelli, Martina Ghetti, Roberta Napolitano, Gabriella Chirumbolo, Giovanni Marconi, Cristina Papayannidis, Stefania Paolini, Chiara Sartor, Giorgia Simonetti, Timothy J. Yen, Giovanni Martinelli

**Affiliations:** 1 Istituto Scientifico Romagnolo per lo Studio e la Cura dei Tumori (IRST) IRCCS, 47014 Meldola, Italy; andrea.ghellilusernadirora@irst.emr.it (A.G.L.D.R.);; 2 Department of Experimental, Diagnostic and Specialty Medicine, Institute of Hematology “L. e A. Seràgnoli”, University of Bologna, 40138 Bologna, Italy; 3 AI Therapeutics, Guilford, CT 06437, USA; 4 Cancer Biology Program, Fox Chase Cancer Center, Philadelphia, PA 19111-2497, USA

**Keywords:** acute lymphoblastic leukemia, synergism, DNA damage response, WEE1, CHK1

## Abstract

Introduction: Screening for synthetic lethality markers has demonstrated that the inhibition of the cell cycle checkpoint kinases WEE1 together with CHK1 drastically affects stability of the cell cycle and induces cell death in rapidly proliferating cells. Exploiting this finding for a possible therapeutic approach has showed efficacy in various solid and hematologic tumors, though not specifically tested in acute lymphoblastic leukemia. Methods: The efficacy of the combination between WEE1 and CHK1 inhibitors in B and T cell precursor acute lymphoblastic leukemia (B/T-ALL) was evaluated in vitro and ex vivo studies. The efficacy of the therapeutic strategy was tested in terms of cytotoxicity, induction of apoptosis, and changes in cell cycle profile and protein expression using B/T-ALL cell lines. In addition, the efficacy of the drug combination was studied in primary B-ALL blasts using clonogenic assays. Results: This study reports, for the first time, the efficacy of the concomitant inhibition of CHK1/CHK2 and WEE1 in ALL cell lines and primary leukemic B-ALL cells using two selective inhibitors: PF-0047736 (CHK1/CHK2 inhibitor) and AZD-1775 (WEE1 inhibitor). We showed strong synergism in the reduction of cell viability, proliferation and induction of apoptosis. The efficacy of the combination was related to the induction of early S-phase arrest and to the induction of DNA damage, ultimately triggering cell death. We reported evidence that the efficacy of the combination treatment is independent from the activation of the p53-p21 pathway. Moreover, gene expression analysis on B-ALL primary samples showed that Chek1 and Wee1 are significantly co-expressed in samples at diagnosis (Pearson *r* = 0.5770, *p* = 0.0001) and relapse (Pearson *r*= 0.8919; *p* = 0.0001). Finally, the efficacy of the combination was confirmed by the reduction in clonogenic survival of primary leukemic B-ALL cells. Conclusion: Our findings suggest that the combination of CHK1 and WEE1 inhibitors may be a promising therapeutic strategy to be tested in clinical trials for adult ALL.

## 1. Introduction

Currently, the standard therapeutic regimens for adult acute lymphoblastic leukemia (ALL) patients still involve DNA-damaging agents such as conventional chemotherapy agents (Hyper-CVAD) [1] with or without the use of specific kinase inhibitors for particular ALL sub-types. Although the initial overall response rate is high (90%) in adult ALL patients, a large percentage of them frequently relapse. Thus, there is a strong need to improve the efficacy of treatments or to identify novel therapeutic strategies that lead to increased durability. For several years different research groups have focused on the biological pathways underlying cancer cell response to DNA damaging therapeutics [2,3]. They discovered that all eukaryotic cells respond to DNA damage by activating DNA damage response (DDR) pathways which consist of cell cycle checkpoints, DNA repair and apoptosis mediators. The overall function of DDR pathways is to prevent cells from initiating cell division with damaged DNA, which would otherwise compromise the DNA of the daughter cells. If DDR pathways play a tumor suppressor role in normal cells, their excessive activation in cancer cells has been associated with increased genetic instability and chemo-resistance, due to the upregulation of DNA-repair mechanisms. Therefore, components of the DDR pathway are interesting targets of cancer cells. The inhibition of cell cycle checkpoint regulators, such as CHK1, CHK2 and WEE1 kinases, has become a promising strategy to kill cancer cells mostly by enhancing the toxicity of DNA-damaging agents [4,5,6,7]. Several studies showed that all three kinases are involved in the regulation of both intra-S and G2/M checkpoints [8,9,10,11,12,13]. Biologically, these three kinases cooperate at different levels to regulate the activity of common substrates such as the CDK2-CYCLIN E (S phase) and CDK1-CYCLIN A/B (G2/M phase) complexes [14]. It has been reported that the inhibition of CHK1, CHK2 or WEE1, as monotherapy or in combination with DNA damaging agents, reduces cell viability and induces apoptosis in preclinical models of ALL [15,16,17,18,19,20]. Recently, an interspecies study highlighted the synthetic lethal interaction between CHK1/CHK2 and WEE1 [21]. The mechanism of action is based on the drug-to-drug synthetic lethality, in which the concomitant inhibition of two independent pathways results in a synergic inhibition of the cell viability. Recent studies on different tumors, including onco-hematological models, showed that the simultaneous inhibition of CHK1 and WEE1 causes synergic inhibition of tumor growth and induction of apoptosis [20,22,23,24,25]. However, none of them focused on ALL. Since WEE1 and CHK1 are important for ALL cell viability and their inhibition results in the collapse of replication forks and impairs cell cycle progression [26,27], we set out to explore this potential therapeutic strategy on ALL cell lines and primary leukemic cells, highlighting the mechanistic cooperativity of the two inhibitors.

## 2. Methods

### 2.1. Drugs and Cell Lines

AZD-1775, PF-00477736 and methotrexate were purchased from MedChemExpress (Monmouth Junction, NJ, USA). Clofarabine was obtained from Sigma-Aldrich (Darmstadt, Germany). Both NALM-6 and RPMI-8402 were cultured in complete RPMI-1640 (Invitrogen, Waltham, MA, USA) medium containing 1% l-glutamine (Sigma-Aldrich), penicillin (100 U/mL, Gibco, Waltham, MA, USA) and streptomycin (100 μg/mL, Gibco) and 10% fetal bovine serum (Gibco).

### 2.2. Viability and Combination Index Analysis

The cell viability analysis of AZD-1775 and PF-00477736 in monotherapy was performed as previously reported [28,29]. For combination index assays, cells were treated simultaneously with increasing concentration of AZD-1775 and PF-00477736 for 24, 48 and 72 h and the reduction of the cell viability was performed using CellTiter 96^®^ AQueous One Solution Cell Proliferation Assay (MTS, Promega, Madison, WI, USA). The additive, synergistic, and antagonistic effect of the drug combinations was evaluated according to the Chou-Talaley equation [30], using Compusyn Software (ComboSyn Incorporated, Paramus, NJ, USA). Based on developer instructions we defined: synergism where CI < 1; additivity where CI = 1; antagonism where CI > 1. To evaluate the effect of the combination of the cell proliferation, cells were seeded in 6-well plates at a concentration of 0.2 × 10^6^ cells/mL and treated with the two compounds in single agent or in combination (DAY 0). Cells were counted every 24 h for 6 days of continuous drug exposure. Proliferation assays were performed in triplicate and independent experiments were performed at least 3 times.

### 2.3. Cell Cycle and Apoptosis Analysis

The induction of cell apoptosis was evaluated using Annexin V-FITC and Propidium Iodide (PI) staining according to the manufacturer’s instructions (Roche, Basel, Switzerland). Cells were seeded for 24 h at 37 °C in 12-well plates at 0.5 × 10^6^ cells/mL with AZD-1775 (both cell lines: 185 nM) and/or PF-00477736 (NALM-6: 250 nM; RPMI-8402: 25 nM). A minimum of 10,000 cells was evaluated for every experimental condition. Statistical analyses were obtained using the mean percentage of Annexin V-PI-positive cells and standard error measurements were calculated from at least three independent experiments. Cell cycle analyses were performed using the same experimental conditions reported above for the apoptosis analysis. After 24 h of incubation, cells fixed using 70% ethanol and stored at −20 °C for 24 h for further cytometry analysis. To perform cell cycle analysis, ethanol was removed by PBS wash and the cells were stained with PI staining mix for 30 min at 37 °C. Cell cycle phases were identified and quantified using Flowing software (Cell Imaging Core, Turku Centre for Biotechnology, Turku, Finland) and ModfiT software (Verity Software House, Topsham, ME, USA).

### 2.4. BrdU Incorporation Assay

In order to quantify the proliferation index, NALM-6 and RPMI-8402 cell lines were stained using BrdU staining (according to datasheet instruction). Cells were seeded at 0.5 × 10^6^ cells/mL on a 6 wells plate and treated with AZD-1775 (both cell lines: 185 nM) and PF-00477736 (NALM-6: 250 nM; RPMI-8402: 25 nM) for 22 h at 37 °C. After 22 h of incubation, cells were incubated with BrdU labeling solution for further 2 h and then fixed. Samples were analyzed using luminomer (Infinite 200 PRO NanoQuant Microplate Reader, Tecan, Männedorf, Switzerland). Row data (RLU, relative luminescence unit) were normalized and expressed as a percentage of the control cells.

### 2.5. Clonogenic Assay and Morphological Analysis

Primary mononuclear cells isolated from the bone marrow (BM) of adult BCR/ABL1-positive/-negative B-ALL patients (*n* = 7) at diagnosis or relapse (not paired) were seeded at 0.75 × 10^5^ cells/well in methylcellulose-based medium (StemMACS HSC-CFU complete with Epo; Miltenyi Biotec, Bergisch Gladbach, Germany). The study was approved by the local Ethical committee (n. 112/2014/U/Tess). Informed consent was obtained in accordance with the Declaration of Helsinki. Cells were incubated with PF-00477736 (0.1 μM) with or without AZD-1775 (0.1 μM) for 14 days at 37 °C. Colonies were counted and the reduction of the clonogenic capacity was calculated as a percentage of the number of colonies in the control (number of colonies in the treatment/number of colonies in the control × 100). To better define the effect of the in vitro treatments on BM hematopoietic precursors and on primary leukemic B-ALL cells, at the end of the clonogenic assays (*n* = 3), the colonies were harvested, washed in PBS to remove the methylcellulose, seeded on poly-D-lysine-coated cover-slides and stained with MC Grunwald & Giemsa solution (J.T.Baker, ThermoFisher Scientific, Waltham, MA, USA). An average number of 300 cell/experimental condition was evaluated to quantify the number of cells.

### 2.6. Quantitative PCR of CHK1, CHK2 and WEE1 in Primary B-ALL Samples

Total RNA was extracted using simply RNA Blood Kit (Promega) from primary leukemic cells isolated from the BM of the seven B-ALL cases used for the above described clonogenic assays. One µg of total RNA was used as template for reverse transcription according to the SuperScript IV protocol (ThermoFisher Scientific). The equivalent cDNA was analyzed by Taqman Gene Expression assays-single tube assays (ref. 4331182- Applied Biosystems, Foster City, CA, USA) for CHK1, CHK2 and WEE1 expression, using GUS-β (Beta-Glucuronidase) as control gene (ENF1102 5′ GAAAATATGTGGTTGGAGAGCTCATT3′, ENR1162 5′CCGAGTGAAGATCCCCTTTT TA3′, ENPr1142Fam CCAGCACTCTCGTCGGTGACTGTTCA-Joe). All reactions were performed in triplicate (both genes of interest and CG) on a Taqman 7900HT real-time PCR machine (ThermoFisher Scientific). The relative gene expression values for each gene of interest were calculated by ΔΔCT method following the recommendations provided by thermofisher.com/qpcreducation on RQ Manager application (SDS 2.4 software, Applied Biosystems). Furthermore, the differential expression value between CHK1, CHK2 and WEE1 genes at disease state (diagnosis or relapse) was determined by fold change formula 2-ΔΔCT.

### 2.7. Immunoblotting

Immunoblotting analyses were performed on cells previously incubated with cell lysis buffer (#9803s, Cell Signaling Technology Danvers, MA, USA) for 30 min. Electrophoresis was performed using Mini-Protean TGX stain-free precast gels, blotted to nitrocellulose membranes (Bio-Rad Trans-blot turbo transfer pack, Bio-Rad, Hercules, CA, USA). After blocking for 1h at room temperature in PBS, with 0.1% (*v*/*v*) Tween 20 (PBST) and 5% milk (*w*/*v*), the membranes were incubated over night at 4 °C with primary antibody diluted 1:1000 in PBST with 0.5% milk or Bovine Serum Albumin (BSA). Membranes were then washed 2 times with PBST, followed by incubation for 1–2 h at room temperature with anti-mouse IgG (NA931V) or anti-rabbit IgG (NA934V) secondary antibody (1:10000 and 1:7000 respectively) from GE Healthcare (Chicago, IL, USA). Membranes were then washed 3 times with PBST and all the antibodies were detected using the enhanced chemiluminescence kit ECL (GE) and the compact darkroom ChemiDoc-It (UVP).

The primary antibodies used are listed following: CHK1 (#2345S), pCHK1 (Ser317) (#2344S), and pCHK1 (ser345) # 2348S), CHK2(#2662S), pCHK2(#2661S), CDK1(# 28439S), CDK2 (#9112S), pCDK1 (Tyr15) (#4539S), CDC25C (#4688), CDC25A (#3652), CCNE (# 20808S), CDKN1A (#2947), γH2A.X (Ser139) (#2577S), WEE1 (#4936S), ATM (#2873S), pATM (#5883S), ATR (#2790S), and pATR (#2853S) from Cell Signaling. Antibody against CCNB1(ab18250) and CCNA1(ab53699) were purchased from Abcam (Cambridge, UK). Antibody to p53 (NBP2-50538) was purchased from Novus Biologicals (Centennial, CO, USA). Antibody to β-actin has been used as internal control and was purchased from S igma Aldrich (St. Louis, MO, USA). For each protein the intensity of the bands was analyzed using ImageJ software (National Institutes of Health, Bethesda, MD, USA). The relative quantity of each protein was calculated as a ratio between the relative intensity of the protein of interest in the treated samples divided by the relative intensity of the same protein in the controls.

### 2.8. Gene Expression Profiling

CHK1/CHK2 and WEE1 gene expression level was performed on total RNA isolated from BM and peripheral blood (PBL) leukemic cells of 51 adult ALL patient samples using Affymetrix GeneChip Human Transcriptome Array 2.0 (ThermoFisher scientific) following manufacturers’ instructions. In detail the gene expression profile was performed on 33 BCR-ABL1-negative (23 at the time of diagnosis and 6 unpaired relapse) and 25 BCR-ABL1-positive (16 diagnosis and 8 unpaired relapse) samples. The normalized mRNA level was obtained using the Gene Level SST-RMA algorithm before row data normalization with Expression Console Software 1.4 (ThermoFisher scientific).

### 2.9. Statistical Analysis

Statistical analyses were performed using Graphpad5 software (GraphPad Inc., San Diego, CA, USA). For the biological experiments the data were presented as the mean ± standard deviation (SD) obtained from the results of at least three independent biological replicates. Statistical significance between groups was evaluated using Two-tailed *t*-test or One-way ANOVA test between groups. Pearson’s correlation coefficient was used to measure the positive or negative co-expression of two genes. In the figures statistical significance was represented as asterisks and in detail: *p* < 0.05 one asterisk (*); *p* < 0.01 two asterisks (**); *p* < 0.001 three asterisks (***).

## 3. Results

### 3.1. The Simultaneous Inhibition of CHK1, CHK2 and WEE1 Impairs ALL Cell Lines Viability and Triggers Apoptosis

**Figure 1:**
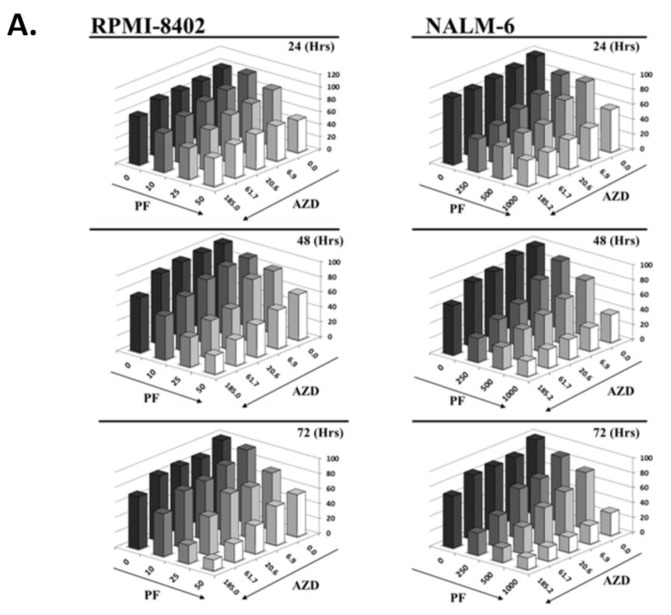

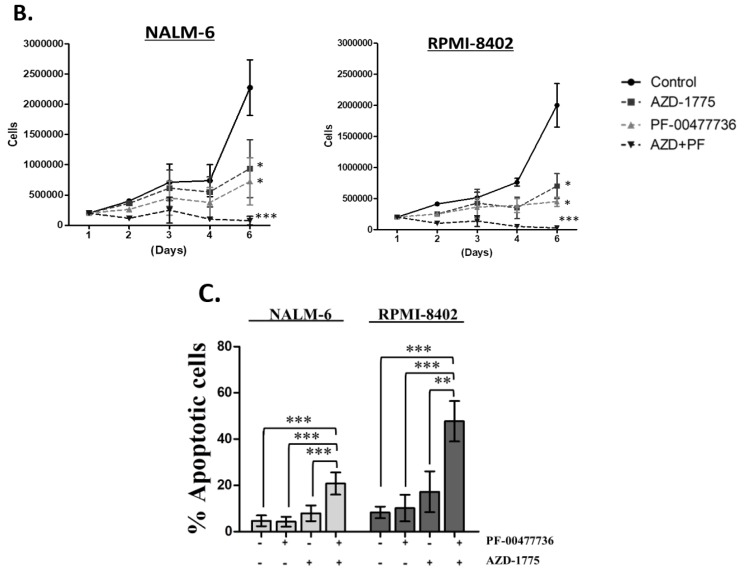
The simultaneous inhibition of CHK1/CHK2 and WEE1 synergizes in term of reduction of cell viability and induction of apoptosis in ALL cell lines. (**A**) Cell viability of RPMI-8402 and NALM-6 cell lines treated simultaneously with increasing concentration of PF-00477736 (nM) and AZD-1775 (nM) for 24, 48 and 72 h. Abbreviations PF = PF-00477736, AZD = AZD-1775. (**B**) Growth curve analysis of RPMI-8402 and NALM-6 cell lines treated for 24 h with AZD-1775 (185 nM; dark grey full square) and PF-00477736 (25 and 250 nM respectively; light gray full triangle). In the graph AZD-1775 in combination with PF-00477736 is named AZD + PF(black full triangle). (**C**) Apoptosis analysis on RPMI-8402 and NALM-6 cell lines treated for 24 h with AZD-1775 (185 nM) and PF-00477736 (25 and 250 nM respectively). In the figures statistical significance was represented as asterisks and in detail: *p* < 0.05 one asterisk (*); *p* < 0.01 two asterisks (**); *p* < 0.001 three asterisks (***).

To test the efficacy of CHK/CHK2 and WEE1 inhibition we initially used ALL cell lines. Recently, we published on the effectiveness of targeting CHK1/CHK2 kinase as a single agent in ALL models [29] and based on that, the most sensitive (RPMI-8402) and the less sensitive (NALM-6) cell lines to the CHK1/CHK2 inhibitor PF-00477736 were selected as models for the present study. Cells were treated simultaneously with increasing concentrations of AZD-1775 (185 to 6 nM, 1:3 dilution series) and with PF-00477736 (RPMI-8402: 10, 25 and 50 nM; NALM-6 250, 500 and 1000 nM) for 24, 48 and 72 h. In line with published data [29,31,32,33,34], the simultaneous inhibition of the three target kinases resulted in synergistic reduction of cell viability (Figure 1A). Chou-Talalay analysis showed that the combination index (C.I.) values were between 0.17 (strong synergism) and 0.9 (slight synergism) (Table S1). We next confirmed that the reduction of cell viability correlated with a statistically significant reduction of cell growth (Figure 1B) and induction of apoptosis (Figure 1C) specifically in samples treated with PF-0477736 plus AZD-1775 (hereafter PF + AZD). The effect of the combination in terms of induction of apoptosis was greater in RPMI-8402 cells (47% apoptotic cells) compared with NALM-6 cells (27% apoptotic cells). To understand the difference in the apoptotic response, we evaluated several intrinsic characteristics of the two cell lines which may mediate the responsiveness to the combination treatment. In relation to the three target kinases, the two cell lines were comparable in terms of copy number alteration (Figure S1A), transcript level (Figure S1B) but not protein expression. Notably, the basal protein expression of WEE1 was higher (4-fold) in RPMI-8402 in comparison to NALM-6 cell line suggesting that the former cell line may have greater dependency on WEE1.

### 3.2. The Inhibition of CHK1, CHK2 and WEE1 Kinases Affect Early S-phase Regulation Cells

**Figure 2:**
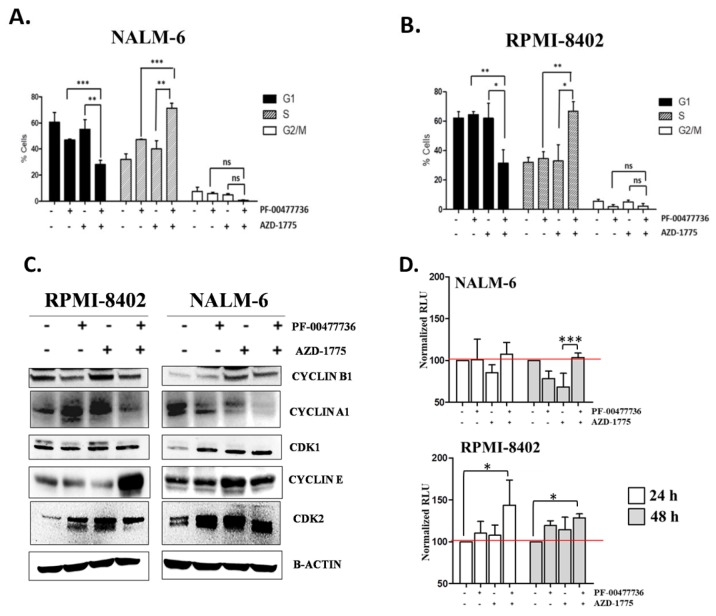

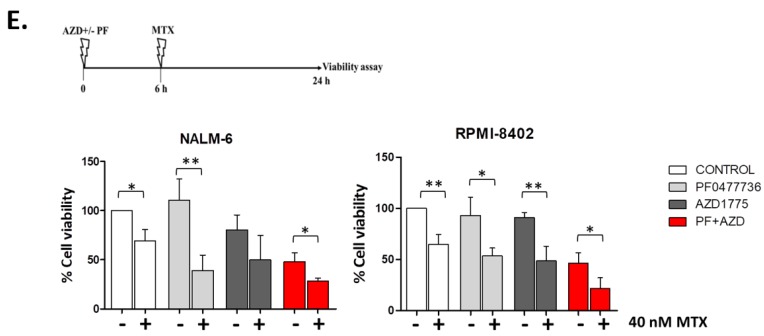
PF-00477736 in combination with AZD-1775 causes early S-phase arrest. (**A**) Cell cycle analyses of NALM-6 cell lines treated simultaneously with subtoxic concentration AZD-1775 (185 nM) and PF-00477736 (250 nM) for 24 h. The histograms show the percentage of cells in a specific cell cycle phase. (**B**) Cell cycle analyses of RPMI-8402 cell lines treated simultaneously with subtoxic concentrations of AZD-1775 (185nM) and PF-00477736 (25 nM) for 24 h. The histograms show the percentage of cells in a specific cell cycle phase. (**C**) Western Blot analyses of RPMI-8402 and NALM-6 cell lines treated for 24 h with AZD-1775 (185 nM) and PF-00477736 (25 and 250 nM respectively). β-actin was used for loading normalization. For relative quantification of each protein see Figure S1C and for whole western blot images see Figure S4. (**D**) The graph represents the normalized RLU (relative light unit) of NALM-6 and RPMI-8402 treated with AZD-1775 (185 nM) and PF-00477736 (25 and 250 nM respectively) for 24 and 48 h. the experiments were performed in triplicates. (**E**) Viability analysis of NALM-6 and RPMI-8402 cell lines treated with AZD-1775 (185 nM) and PF-00477736 (250 and 25 nM, respectively) for 6 h and then with MTX (40 nM) for 18 h. Above the histograms is schematically represented the experimental procedure for the drug combination studies. The flash lighting points when the drugs were added to the cell culture. In the figures statistical significance was represented as asterisks and in detail: *p* < 0.05 one asterisk (*); *p* < 0.01 two asterisks (**); *p* < 0.001 three asterisks (***).

To understand the consequences of CHK1/CHK2 and WEE1 inhibition on cell cycle progression, cells were analyzed for DNA content using PI staining. The PF + AZD treatment significantly decreased the number of cells in G1 and significantly increased the number of cells in S phase, thereby confirming the combination impaired cell cycle progression (Figure 2A,B). Immunoblotting analysis revealed that the combination treatment stabilized the CDK2-CYCLIN E complex, master regulator of the G1/S transition [35,36], resulted in the increase of CYCLIN E and CDK2 levels as compared to the single agents and controls. In detail, CDK2 level was increased by the treatments reaching the higher level in the combination treatment in both cell lines. Similar results were found for CYCLIN E protein level on RPMI-8402 cells. In NALM-6 cell, no difference was found between combination treatment and AZD alone in term of CYCLIN E1 level (Figures S1C and S4). In line with the cell cycle data, no significant difference was seen in the levels of CDK1-CYCLIN B complex (master regulator of the G2/M transition) between single and PF + AZD treatments. In both cell lines, PF + AZD treatment reduced the amount of CYCLIN A1 in comparison to the single treatment and to the controls (Figure 2C, Figures S1C and S4). To further investigate the effect of the combination on cell cycle progression, cells treated with PF, AZD, and their combination for 24 and 48 h, were incubated with BrdU to directly assess the effects on DNA replication. For NALM-6 cells, treatment with inhibitors for 24 h did not affect BrdU incorporation when compared to untreated controls. At 48 h, both single treatments reduced BrdU incorporation suggesting inhibition of replication. However, the combination treatment restored replication to control levels, suggesting overriding of the DNA damage checkpoint by the single agents. In RPMI-8402 cells, single treatment slightly elevated BrdU incorporation relative to control at both 24 and 48 h. Unlike NALM-6 cells, treatment with the PF + AZ combination for 24 h significantly increased BrdU incorporation in comparison to control and single agent. For samples treated with single or the combination for 48 h, BrdU incorporation was elevated when compared to untreated control. These results suggest that the inhibitors do not inhibit DNA replication, and is consistent with the increase in S phase cells (Figure 2D). Finally, to further evaluate the potentials of this new therapeutic strategy, ALL cell lines were treated with methotrexate (MTX) after being exposed to PF-00477736 and/or AZD-1775. MTX is a selective inhibitor of folic acid and it has been showed to reduce both purine and pyridine pools [37]. The scientific rationale for this drug regimen was to exploit the S-phase enhancement of the combination to increase the replicative stress and to enhance replicative forks collapse. Interestingly, the inhibition of CHK1/CHK2 or WEE1 in single agent or in combination enhanced the cytotoxicity of MTX (Figure 2E).

### 3.3. The Combination AZD-1775 and PF-00477736 Triggers the S Phase Checkpoint and Induce DNA Damage

**Figure 3:**
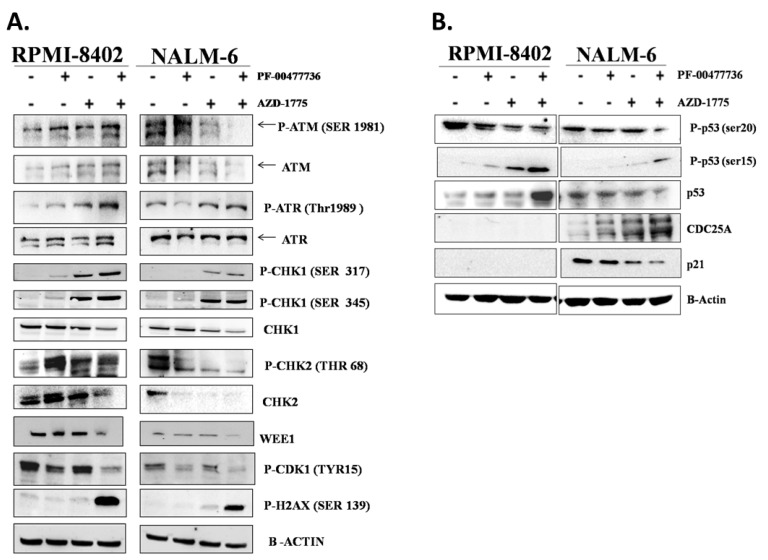
The combination triggers the DDR pathway and induces DNA damages independently for p53 functionality. (**A**) Western Blot analyses of RPMI-8402 and NALM-6 cell lines treated for 24 h with AZD-1775 (185 nM) and PF-00477736 (25 and 250 nM respectively). β-actin was used for loading normalization. For relative quantification of each protein see Figure S2 and for whole western blot images see Figure S5. (**B**) Western Blot analyses of RPMI-8402 and NALM-6 cell lines treated for 24 h with AZD-1775 (185 nM) and PF-00477736 (25 and 250 nM respectively). β-actin was used for loading normalization. For relative quantification of each protein see Figure S3A and for whole western blot images see Figure S6.

We then analyzed the effects of the simultaneous inhibition of CHK1, CHK2 and WEE1 on the DDR pathways and the induction of DNA damage. Single agent treatments only weakly induced the phosphorylation of H2AX (ser139), a bona fide marker of DNA damage, in comparison with the combination treatment (Figure 3A, Figures S2 and S5). Sub-toxic concentrations of AZD-1775 alone significantly activated CHK1 kinase (phosphorylation at ser345 or ser317)-dependent response, which was also observed in the combination treatment. The combination treatment did not increase phosphorylation in ser345, necessary for the nuclear localization of CHK1, in both the cell lines. Moreover, the drug combination significantly increased ser317 phosphorylation which is one of the crucial events after stalled replication forks and is specifically mediated by ATR kinase [38]. Taking into account total levels of CHK1, the amount of phospho-CHK1 ser317 appear to be significantly highest in the combination treatment. Consistent with their ability to override the DNA damage checkpoint arrest, the inhibition of both CHK1/CHK2 and WEE1 additively reduced the amount of phospho-CDK1 (Tyr15) in both cell lines [39] (Figures S2 and S5). Changes were also observed in CHK2 activity and stability in the PF + AZD treated with a different outcome among the two cell lines. In RPMI-8402 cells the single treatments increased the amount of phospho-CHK2 while the co-treatment did not further increase the levels of phospho-CHK2. However, when the total levels of CHK2 is taken into account, the amount of phospho-CHK2 appear to be highest in the combination treatment. In NALM-6 cells, the basal level of phospho-CHK2 in untreated cells is high. Both single and co-treatment reduced the level of phospho-CHK2. This however, is due to the dramatic reduction in the total levels of CHK2 after treatment with inhibitors. The heterogeneous effect of both single treatments and combination of CHK2 was likely related to the protein expression of the up-stream activator, ATM. Indeed, in RPMI-8402 cells the combination treatment additively increased the amount of both basal and phospho-ATM (ser1981) while decreased them in NALM-6 cells (Figure 3A, Figures S2 and S5).

### 3.4. The Effect of the Combination Is Independent the Activity of p53 in the Regulation of Cell Cycle

TP53 is mutated in 16% of adult ALL patients and it has been showed to correlate with poor prognosis [40,41,42]. P53 is phosphorylated during cell cycle checkpoint activation in response to DNA damage by ATM/ATR [43,44,45] and by CHK1/CHK2 [46,47]. We therefore studied the role of p53 activity in cell cycle regulation following drug treatments. AZD + PF treatment increased the amount of phospho-p53 (ser15) but not of phospho-p53 (ser20) in both cell lines. These findings highlighted that p53 is activated in both cell lines in response to DNA damage by the activity of ATR and ATM (increase of phospho-p53 ser15), but its complete activation, necessary for its transcriptional activity, was compromised (decrease of phospho-p53 ser20) [48,49]. In RPMI-8402 cells, which are *TP53*-mutated (NM_000546.5: c.817C>T; p.Arg273Cys), the combination also promoted a significant increase in the total level of p53 relative to control and single agent treatments. The missense mutation, located in the DNA binding domain, prevents the activation of the downstream DNA damage response pathway. This explains the absence of activation of crucial downstream targets like p21 [50,51] and CDC25A [52,53,54] in all samples. Conversely, in the TP53 proficient NALM-6 cells, the inhibitor combination reduced the basal amount of p53. This then leads to loss of p21 expression which in turns accounts for the increased expression of CDC25A (Figure 3B, Figures S3A and S6). It has been also shown that p53 promotes CDC25A down-regulation in response to DNA damages, through the activation of the transcriptional repressor ATF3 [52].

### 3.5. CHK1 and WEE1 Are Co-Expressed in ALL Primary Cells

**Figure 4:**
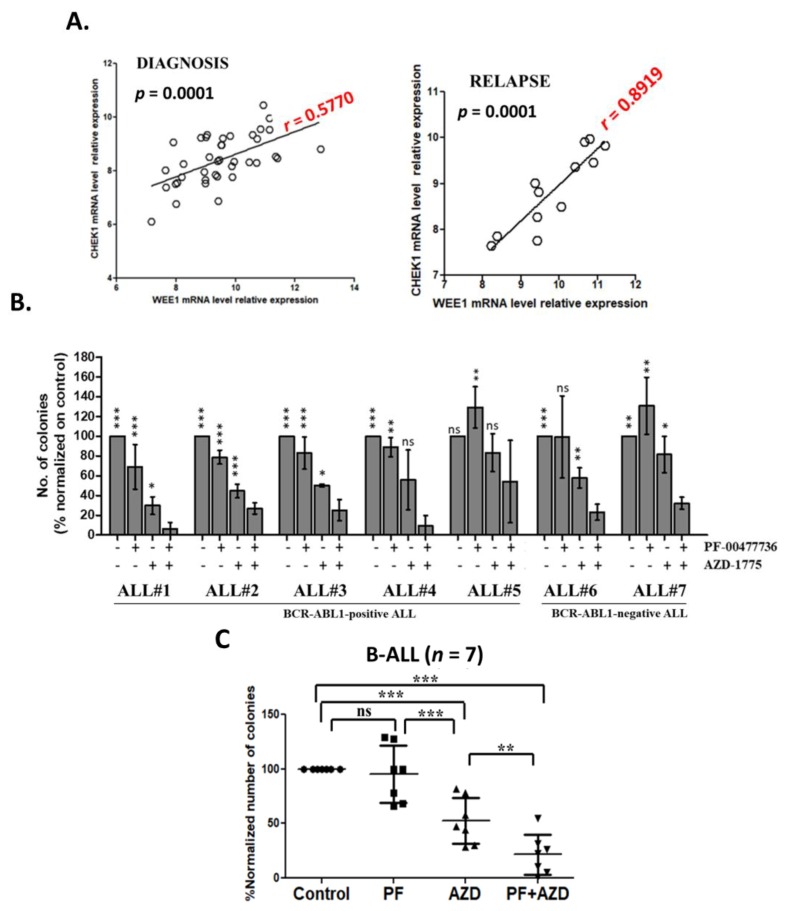
The inhibition of CHK1, CHK2 e WEE1 compromises primary leukemic B-ALL clonogenic capacity. (**A**) Dot plot showing the Pearson correlation (*r* value) of *Wee1* and *Chek1* relative mRNA level in primary leukemic ALL samples at diagnosis (*n* = 39) and at relapse (*n* = 14). On the top of each dot plot is reported the statistical significance of the analysis and on the linear regression is reported in red the Pearson correlation value (*r*). (**B**) Clonogenic assays of primary leukemic cells isolated from the bone marrow of adult B-ALL patients (*n* = 7). The columns represent the normalized number of colonies in the treated samples in relationship to the control. The asterisks in the graph represent how the effect of the combined treatment in the reduction of the number of colonies is significantly different from the single treatments and from the un-treated control. (**C**) Scatter plot representing the mean of 7 independent experiments. In the graph AZD-1775 in combination with PF-00477736 is named AZD + PF. In the figures statistical significance was represented as asterisks and in detail: *p* < 0.05 one asterisk (*); *p* < 0.01 two asterisks (**); *p* < 0.001 three asterisks (***).

To further evaluate whether CHK1/CHK2 and WEE1 could be therapeutic targets in ALL, we first assessed the level of expression of these kinases in primary leukemic ALL samples. We previously showed that all three kinases are highly expressed in ALL samples in comparison to normal mononuclear cells [28,55] but we did not investigate their level of co-expression. Here, in primary leukemic blasts isolated from the bone marrow (BM) or the peripheral blood (PB) of adult B-ALL patients (for patient characteristics see [28]) at diagnosis (*n* = 39) and relapse (*n* = 14; not paired samples) we observed that *Wee1* and *Chek1* transcripts are co-expressed at diagnosis (Pearson *r* = 0.5770, *p* = 0.0001) and strongly co-expressed at relapse (Pearson *r*= 0.8919; *p* = 0.0001) (Figure 4A). Based on the results of the functional studies on ALL cell lines, we then evaluated the co-expression of different elements involved in the regulation of the DDR pathways (Table S2). The results of the analysis showed high level of positive co-expression between *Chek1*/*Chek2*/*Wee1* and several genes involved in the DDR pathway (Tables S3 and S4).

### 3.6. Inhibition of CHK1/2 and WEE1 Affect the Clonogenic Capacity of Primary ALL Cells

Primary mononuclear cells isolated from the BM of B-ALL patients (*n* = 7, Table S5) at diagnosis or relapse (not paired) were seeded in methylcellulose-based medium and treated for 14 days with PF-00477736 (0.1 μM) with or without AZD-1775 (0.1 μM). Based on the average number of colonies counted in the single treatments and in the combined treatment, the clonogenic assays are consistent with the effect of the drug combination in cell viability assays. On primary leukemic cells the effect of the combination in term of reduction of number of colonies resulted additive (Figure 4B,C). We sought to correlate the in vitro sensitivity to the combination with the patient-relative expression of the three targets genes, we performed quantitative PCR analyses. However, no significant association was identified (Figure S3C). To better define the effect of the in vitro treatments on BM hematopoietic precursors the colonies were harvested after 14 days and stained with MC Grunwald & Giemsa solution. According to the routine diagnostic procedure [56], the slides were analyzed for the count of hematopoietic precursors, primary leukemic blasts and hematopoietic cells based on cell morphology. The morphological staining confirmed a reduction of leukemic cells’ count in the combinations compared with the single agent treatments and the control (Figure S3C). Moreover, the number of promyelocytes was not affected, while monocytes/macrophages showed a trend towards increased amount, while the number of undefined precursors was significantly reduced by the drug (Figure S3C).

## 4. Discussion

The identification of novel therapeutic strategies for the treatment of ALL patients who relapse after standard of care therapy is an urgent clinical need. Although the initial response to treatments is generally positive, a large percentage of patient relapses or becomes refractory to conventional therapies and the identification of novel functional and molecular targets could improve the outcome of ALL patients. Our group, together with many others, is currently focusing on applying the concept of synthetic lethality to selectively target cancer cells. In the scenario of two pathways regulating a crucial biological function, the inhibition of one pathway is compensated by the second pathway. However, the simultaneous inhibition of both pathways results in the complete loss of the biological function. Several genes are involved in the regulation of the cell cycle checkpoints. These genes cooperate in the regulation of common substrate. For example, CDK1 and CDK2 activity is known to be regulated directly by the inhibitory phosphorylation promoted by WEE1 and PKMYT1, and indirectly by CDC25 family proteins. We previously reported the efficacy of selective CHK1/CHK2 or WEE1 inhibitors in disrupting the cell cycle checkpoint control [28,29,31]. Indeed, the inhibition of checkpoint functionality increased the genetic instability to a point of no return. In the present study we speculated that the simultaneous inhibition of CHK1/CHK2 and WEE1 kinases could synergistically impair the functionality cell cycle checkpoint, inducing massive DNA damage and resulting in cell death. Indeed, it has been showed that WEE1 inhibition results in stalled replication forks that can eventually collapse [26]. In turn, this activates CHK1 to presumably help coordinate the repair of these damaged sites. In this scenario, co-treatment with a WEE1 and a CHK1 inhibitor blocks the cells ability to repair the damage, thus inducing apoptosis. This hypothesis was confirmed by the synergistic reduction of cell viability observed in NALM-6 and RPMI-8402 cell lines, along with significant induction of cell death in the samples treated with the drugs combination.

### 4.1. The Inhibition of CHK1/WEE1 Kinases Highlights Potential Therapeutic Strategies Affecting S Phase

Cell cycle analysis showed that the two inhibitors increased the number of cells in S phase while decreasing the number of cells in G1 while no significant effect was seen in the percentage of cells in G2/M phase. This may be due to the low number of cells in this phase in both cell lines. To characterize the effect of PF-0477736 with AZD-1775 on cell cycle, samples were analyzed for the expression of CDK-Cyclin complexes. Our result showed that in both cell lines the co-treatment synergistically increased CDK2 and/or Cyclin E1 protein expression. Therefore, we speculate that the increase of CDK2/Cyclin E1 expression marks the action of the combination in the early S phase. Indeed, CDK2/Cyclin E1 complex promotes the assembly of the pre-replication complex (CDC6/MCM2-7) on the origin of replications during the transition from G1 to S phase [36,57]. Immunoblotting analysis also highlighted the potential cytotoxic effect of the combination. Indeed, in both cell lines a synergistic induction of γH2AX was seen in the samples treated with PF-0477736 and AZD-1775, consistent with the notion that enhanced DNA damage occurs through induction of break sites and block of repair. The induction of DNA damage was also indicated by the activation of ATR/CHK1 and ATM/CHK2 pathways. The result of the combination studies highlighted that simultaneous inhibition of WEE1 and CHK1 sensitize the ALL cell lines to S-phase specific chemotherapy agents. We have already published regarding the strong synergism using AZD-1775 or other cell cycle checkpoint kinase inhibitors in combination with different chemotherapy agents. Here, we showed that the cytotoxicity PF-0477736 with AZD-1775 can be enhanced using MTX. We hypothesized that the accumulation of cells in S phase may increase the intracellular demand for nucleotides tri-phosphate and thus the use of MTX should results in strong reduction of the cell viability. We believe that similar results may be obtained using antimetabolites such as clofarabine or nelarabine

### 4.2. The Efficacy of the Combination Strategy Is Independent to p53 Functionality in ALL Cell Lines

**Figure 5:**
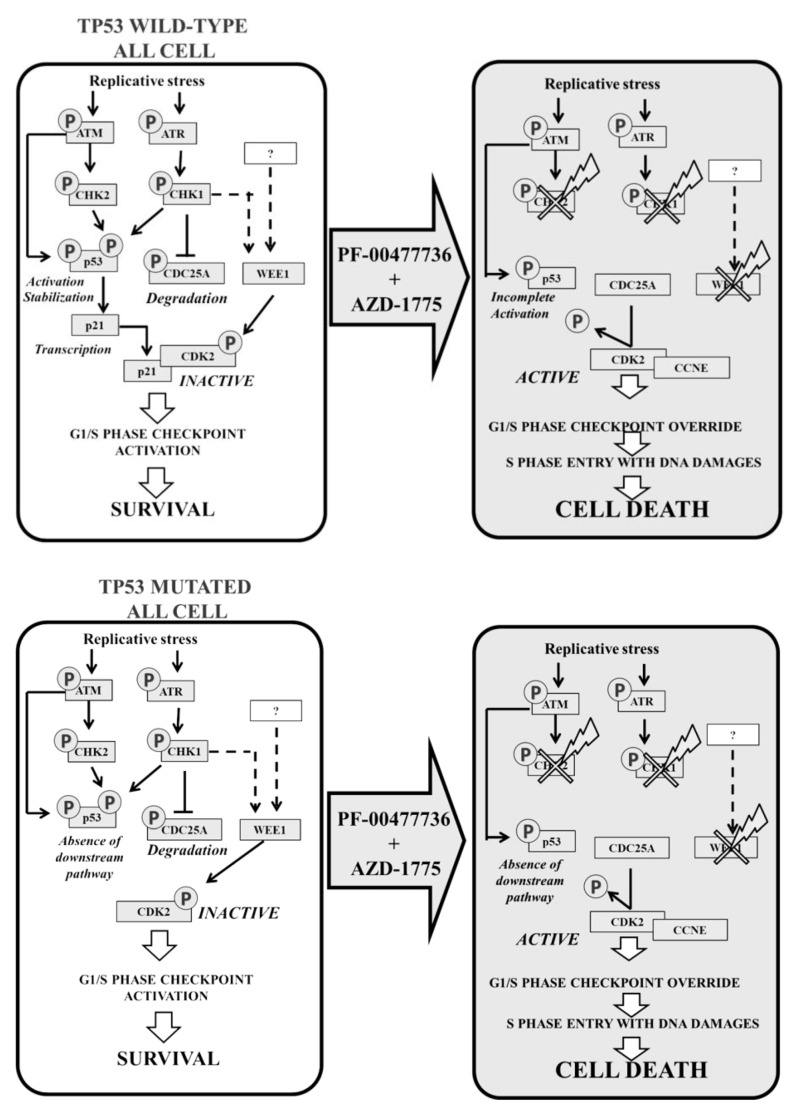
Schematic representation of the combination strategy using PF-0477736 and AZD-1775 in ALL cells.

Interestingly, the effect of the combination was p53- and p21-independent. Indeed, immunoblotting analysis showed that the co-treatment triggered the ATM/ATR pathway as showed by the induction of phospho-p53 (ser15). The inhibition of CHK1/CHK2/WEE1 functionality was confirmed by the reduction of phospho-p53 (ser20). Regarding the basal amount of p53, the combination increased it synergistically in RPMI-8402 while reduced it on NALM-6 cell line. In RPMI-8402 cells, *TP53* mutated, the missense mutation in the DNA binding domain affected the p53 downstream DDR pathway (absence of p21 or CDC25A) resulting in no differences between samples treated with the two inhibitors either in single agent or in combination. Conversely, in NALM-6 cells, p53 proficient, the combination reduced the basal amount of p53, and consequently, the amount of p21 (resulting in a significant increment of CDC25A). In normal conditions p21 would bind to CDK2-Cyclin E1 complex to induce cell cycle arrest (G1/S) [58] (Figure 5).

### 4.3. Limiting the Toxicity of CHK1/WEE1 Inhibition Using Combination Strategies

Currently the use of cell cycle checkpoint or other DDR inhibitors is raising questions about their potential toxicity. Indeed, the inhibition of DDR pathway may expose healthy cells to the risk of accumulation of DNA damages. In our experience, the use of PF-00477736 or AZD-1775 in single agent have limited toxicity on healthy mononuclear cells using ex vivo therapeutic concentrations. Our combination strategies allowed to use sub-toxic concentrations of the two inhibitors to obtain the same in vitro therapeutic effects using one of the two inhibitors in monotherapy. The effect of the co-treatment on ALL primary leukemic cells confirmed the efficacy of the proposed therapeutic approach. Indeed, a significant reduction of primary cell colonies was detected in samples treated with AZD-PF. Despite the overall good response to the drug combination, its efficacy did not correlate with the expression of the three kinases (Figure S3B). However, in patient ALL#5, the poor responsiveness to the combination may correlate with low number of leukemic blasts of the sample (Table S5). Although more accurate analyses should be performed to better define the characteristics of the hematopoietic precursors harvested from the primary colonies, the morphological analysis performed here suggested that the reduction in the number of colonies reflected an effective decrease in the number of ALL primary cells, with a relative low toxicity on healthy hematopoietic precursors. Finally, the gene expression analysis of DDR-related genes highlights novel potential therapeutic option that should be tested in ALL. In our cohort, *Chek1*, *Chek2* and *Wee1* genes are positively co-expressed with other crucial cell cycle regulators and, in particular, with different CDKs (*CDK1*, *CDK2* and *CDK4*). Numerous CDK inhibitors have proven their efficacy in different preclinical and clinical studies against acute leukemias. In this scenario, novel synthetic lethality approaches could be obtained by combining CDK inhibitors with CHK1/CHK2 or WEE1 inhibitor.

## 5. Conclusions

In conclusion, the idea of using targeted drug combinations to concurrently induce DNA damage and compromise DNA repair mechanisms is currently being evaluated in clinical trials in solid tumors (NCT03330847). In this trial cancer patients will be evaluated for the response to AZD-1775 in combination with the PARP-1 inhibitor, olaparib. Patient will be evaluated for their mutational profile in BRCA genes or more generally in DDR-genes. Indeed, it has been showed that the synthetic lethality approach using PARP-1 inhibition is maximum in BRCA1/2 deficient cancer patients. In this scenario, the inhibition of PARP-1 results in the accumulation of DNA damages to a point of no return. The addition of a WEE1 inhibitor to olaparib may force cancer cells to proliferate in the presence of PARP1-mediated DNA damages resulting in a massive induction of cell death. Our data suggest that the combination of CHK1/CHK2 and WEE1 inhibitors may cause a similar functional impairment, thus providing the rationale for its evaluation in clinical trials for adult ALL patients. Our therapeutic approach exploits the intrinsic genetic instability of ALL cells, forcing them to replicate and to progressively accumulate DNA damages during DNA synthesis.

## Supplementary Materials

The following are available online at https://www.mdpi.com/2072-6694/11/11/1654/s1, Table S1: Combination index analyses on RPMI-8402 and NALM-6 cell lines treated for 24, 48 and 72 h with increasing concentration of AZD-1775 and PF-00477736, Table S2: List of analyzed genes that are involved in the DNA damage response pathway, Table S3: Correlation analysis of WEE1, CHK1 and CHK2 expression with DDR genes in primary ALL samples at diagnosis, Table S4: Correlation analysis of primary ALL samples at relapse, Table S5: Primary sample’s characteristics, Figure S1: Copy number, mRNA and protein levels of genes involved in the response to the inhibitors, Figure S2: Histograms showing the relative protein expression of P-ATR(THR1989), ATR, P-ATM(SER1981), ATM, P-CHK1 (SER317), P-CHK1 (SER345), CHK1, P-CHK2 (THR68), CHK2, WEE1, P-CDK1(TYR15) and P-H2AX (SER139) proteins in RPMI-8402 (Black) and NALM-6 (Gray) cells, Figure S3: Effect of the combination in the protein level of ALL cell lines and relative mRNA level and morphological staining of primary B-ALL cells, Figure S4: Whole Western Blot images, Figure S5: Whole Western Blot images, Figure S6: Whole Western Blot images.

## Abbreviations

ALL	acute lymphoblastic leukemia
BM	bone marrow
B-ALL	B-cell precursor acute lymphoblastic leukemia
DDR	DNA damage response
PB	peripheral blood
PI	Propidium Iodide
SD	standard deviation
SER	serine
T-ALL	T-cell precursor acute lymphoblastic leukemia
