# Supplementary material for: Synergism Through WEE1 and CHK1 Inhibition in Acute Lymphoblastic Leukemia
Source: Cancers (Basel). 2019 Oct 25;11(11):1654. doi: 10.3390/cancers11111654 (PMC6895917; doi:10.3390/cancers11111654)
Supplement: Supplementary file 1 [file cancers-11-01654-s001.pdf]

Supplementary Materials: Synergism Through WEE1 and CHK1 Inhibition in Acute Lymphoblastic Leukemia

Andrea Ghelli Luserna Di Rorà, Matteo Bocconcelli, Anna Ferrari, Carolina Terragna, Samantha Bruno, Enrica Imbrogno, Neil Beeharry, Valentina Robustelli, Martina Ghetti, Roberta Napolitano, Gabriella Chirumbolo, Giovanni Marconi, Cristina Papayannidis, Stefania Paolini, Chiara Sartor, Giorgia Simonetti, Timothy J. Yen and Giovanni Martinelli

Table S1. Combination index analyses on RPMI-8402 and NALM-6 cell lines treated for 24, 48 and 72 hours with increasing concentration of AZD-1775 and PF-00477736.

RPMI-8402				NALM-6			
**AZD/PF**	**C.I. (24)**	**C.I. (48)**	**C.I. (72)**	**AZD/PF**	**C.I. (24)**	**C.I. (48)**	**C.I. (72)**
**6.9/25**	0.601	0.61198	0.42268	**6.9/250**	0.34884	0.6052	0.5847
**20.6/25**	0.46692	0.22987	0.42465	**20.6/250**	0.24904	0.39168	0.66437
**61.7/25**	0.27022	0.26433	0.27259	**61.7/250**	0.16627	0.37351	0.40216
**185/25**	0.13081	0.30885	0.1732	**185/250**	0.13558	0.3452	0.36831
**6.9/50**	0.87488	0.35902	0.25588	**6.9/500**	0.7631	0.79851	0.90644
**20.6/50**	0.48613	0.29401	0.22114	**20.6/500**	0.30592	0.66538	0.76636
**61.7/50**	0.25192	0.25492	0.20033	**61.7/500**	0.314	0.60552	0.58861
**185/50**	0.18163	0.24571	0.20489	**185/500**	0.24116	0.52085	0.39227
**6.9/100**	1.73166	0.56524	0.447	**6.9/1000**	0.46692	0.84832	0.78308
**20.6/100**	0.93696	0.47512	0.3262	**20.6/1000**	0.3982	0.7761	0.69849
**61.7/100**	0.4717	0.3794	0.27162	**61.7/1000**	0.31761	0.75357	0.66371
**185/100**	0.32807	0.3876	0.28982	**185/1000**	0.32728	0.70776	0.62144

The additive, synergistic, and antagonistic effect of the combinations was evaluated using viability analyses of two independent experiments and was calculated using Compusyn Software (C.I < 1 synergism; C.I. = 1 additivity; C.I. > 1 antagonism). In the table the drug combination are in bold and the concentrations are expressed in nM.

Table S2. List of representative genes involved in the DNA damage response pathway.

Gene Accession	Gene Symbol	Gene Description	Chromosome	Cytoband
NM_00119974	GADD45A	growth arrest and DNA-damage-inducible, alpha	1	1p31.2
NM_004073	PLK3	polo-like kinase 3	1	1p34.1
NM_001184	ATR	ATR serine/threonine kinase	3	3q23
NM_001018115	FANCD2	Fanconi anemia complementation group D2	3	3p26
NM_001237	CCNA2	cyclin A2	4	4q27
NM_031966	CCNB1	cyclin B1	5	5q12
NM_001790	CDC25C	cell division cycle 25C	5	5q31
NM_001136017	CCND3	cyclin D3	6	6p21
NM_000389	CDKN1A	cyclin-dependent kinase inhibitor 1A (p21, Cip1)	6	6p21.2
NM_001145306	CDK6	cyclin-dependent kinase 6	7	7q21–q22
NM_057749	CCNE2	cyclin E2	8	8q22.1
NM_001261	CDK9	cyclin-dependent kinase 9	9	9q34.1
NM_004235	KLF4	Kruppel-like factor 4 (gut)	9	9q31
NM_001170406	CDK1	cyclin-dependent kinase 1	10	10q21.1
NM_001160124	KLF6	Kruppel-like factor 6	10	10p15
NM_000051	ATM	ATM serine/threonine kinase	11	11q22–q23
NM_053056	CCND1	cyclin D1	11	11q13
NM_001114121	CHEK1	checkpoint kinase 1	11	11q24.2
NM_001143976	WEE1	WEE1 G2 checkpoint kinase	11	11p15.4
NM_001759	CCND2	cyclin D2	12	12p13
NM_001798	CDK2	cyclin-dependent kinase 2	12	12q13
NM_000075	CDK4	cyclin-dependent kinase 4	12	12q14
NM_001111045	CCNA1	cyclin A1	13	13q12.3–q13
NM_000057	BLM	Bloom syndrome, RecQ helicase-like	15	15q26.1
NM_004701	CCNB2	cyclin B2	15	15q22.2
NM_004424	E4F1	E4F transcription factor 1	16	16p13.3
NM_004203	PKMYT1	protein kinase, membrane associated tyrosine/threonine 1	16	16p13.3
NM_007294	BRCA1	breast cancer 1, early onset	17	17q21
NM_000546	TP53	tumor protein p53	17	17p13.1
NM_018955	UBB	ubiquitin B	17	17p12-p11.2
NM_015675	GADD45B	growth arrest and DNA-damage-inducible, beta	19	19p13.3
NM_016270	KLF2	Kruppel-like factor 2	19	19p13.11
NM_001238	CCNE1	cyclin E1	19	19q12
NM_004358	CDC25B	cell division cycle 25B	20	20p13
NM_001005735	CHEK2	checkpoint kinase 2	22	22q12.1

Table S3. Correlation analysis of primary ALL samples at diagnosis.

Gene Symbol	Gene ID	Pearson *r*	95% Confidence Interval	*p* Value (Two-Tailed)
	CHEK2	0.5797	0.3233 to 0.7569	0.0001
	CHEK1	0.577	0.3196 to 0.7551	0.0001
	CDK1	0.5358	0.2650 to 0.7283	0.0004
	BLM	0.5148	0.2378 to 0.7143	0.0008
	CDK2	0.5145	0.2375 to 0.7142	0.0008
	FANCD2	0.4889	0.2049 to 0.6969	0.0016
	CCNB1	0.4774	0.1906 to 0.6892	0.0021
WEE1	CCNA2	0.4651	0.1752 to 0.6808	0.0029
	BRCA1	0.4549	0.1627 to 0.6738	0.0036
	CDC25C	0.422	0.1227 to 0.6509	0.0075
	CCNB2	0.4168	0.1166 to 0.6473	0.0083
	CDC25B	0.4134	0.1125 to 0.6448	0.0089
	**UBB**	**−0.3848**	**−0.6245 to −0.07877**	**0.0156**
	**CCND2**	**−0.4888**	**−0.6969 to −0.2048**	**0.0016**
	**CDK9**	**−0.49**	**−0.6977 to −0.2063**	**0.0015**
	CDK2	0.8316	0.6996 to 0.9087	0.0001
	BRCA1	0.7732	0.6053 to 0.8752	0.0001
	BLM	0.7199	0.5232 to 0.8438	0.0001
	CCNB1	0.7058	0.5021 to 0.8353	0.0001
	CDK4	0.6946	0.4855 to 0.8286	0.0001
	CDC25C	0.643	0.4107 to 0.7969	0.0001
	CDK1	0.6154	0.3721 to 0.7796	0.0001
CHK1	PKMYT1	0.6134	0.3693 to 0.7783	0.0001
	CCNB2	0.589	0.3358 to 0.7628	0.0001
	FANCD2	0.5776	0.3204 to 0.7555	0.0001
	WEE1	0.577	0.3196 to 0.7551	0.0001
	CHEK2	0.5513	0.2853 to 0.7384	0.0003
	TP53	0.5472	0.2800 to 0.7358	0.0003
	CCNA2	0.5319	0.2599 to 0.7256	0.0005
	**GADD45A**	**−0.4458**	**−0.6675 to −0.1515**	**0.0045**
	FANCD2	0.6692	0.4482 to 0.8130	0.0001
	BRCA1	0.6317	0.3949 to 0.7898	0.0001
	CDK1	0.6033	0.3554 to 0.7719	0.0001
	CCNB1	0.5858	0.3314 to 0.7607	0.0001
	BLM	0.582	0.3264 to 0.7583	0.0001
	WEE1	0.5797	0.3233 to 0.7569	0.0001
	CHEK1	0.5513	0.2853 to 0.7384	0.0003
	CCNA2	0.5152	0.2384 to 0.7146	0.0008
	CDC25C	0.5049	0.2252 to 0.7077	0.001
CHK2	PKMYT1	0.4755	0.1882 to 0.6879	0.0022
	CCNB2	0.468	0.1788 to 0.6827	0.0027
	CDK2	0.4521	0.1593 to 0.6719	0.0039
	TP53	0.443	0.1481 to 0.6656	0.0047
	CDK4	0.4236	0.1247 to 0.6520	0.0072
	**CCND2**	**−0.3936**	**−0.6308 to −0.08907**	**0.0001**
	**CDKN1A**	**−0.4032**	**−0.6376 to −0.1004**	**0.0109**
	**UBB**	**−0.4253**	**−0.6532 to −0.1268**	**0.0069**
	**GADD45A**	**−0.4401**	**−0.6635 to −0.1446**	**0.0051**
	**KLF6**	**−0.5453**	**−0.7345 to −0.2774**	**0.0003**

In the table, on the left, is reported which kinase between WEE1, CHK1 and CHK2, has been correlated with the panel of DDR pathway representative genes. In the table it has been reported only Pearson values with a statistic significance $p > 0.01$. In bold are reported the pair of genes that are negatively co-expressed.

Table S4. Correlation analysis of primary ALL samples at relapse.

Gene Symbol	Parameter	Pearson r	95% confidence interval	P value (two-tailed)
WEE1	FANCD2	0.8477	0.5763 to 0.9507	0.0001
	CHEK1	0.8476	0.5759 to 0.9507	0.0001
	PKMYT1	0.8249	0.5233 to 0.9428	0.0003
	BRCA1	0.8071	0.4836 to 0.9366	0.0005
	CDK2	0.7411	0.3468 to 0.9128	0.0024
	CDK1	0.7264	0.3185 to 0.9073	0.0033
	CCNB1	0.7136	0.2944 to 0.9025	0.0042
	BLM	0.7087	0.2852 to 0.9006	0.0046
	CDC25C	0.6728	0.2211 to 0.8869	0.0084
	TP53	0.6405	0.1664 to 0.8741	0.0136
	CDK4	0.6164	0.1275 to 0.8644	0.0189
	CCNE1	0.6927	0.2563 to 0.8946	0.006
CHK1	PKMYT1	0.9236	0.7705 to 0.9759	0.0001
	BLM	0.8733	0.6387 to 0.9594	0.0001
	CDK4	0.8718	0.6347 to 0.9588	0.0001
	WEE1	0.8476	0.5759 to 0.9507	0.0001
	CDC25C	0.8392	0.5562 to 0.9478	0.0002
	CDK2	0.8299	0.5347 to 0.9446	0.0002
	BRCA1	0.7994	0.4667 to 0.9339	0.0006
	FANCD2	0.7952	0.4576 to 0.9324	0.0007
	CDK1	0.7778	0.4208 to 0.9262	0.0011
	TP53	0.7726	0.4101 to 0.9243	0.0012
	CCNB1	0.7485	0.3613 to 0.9155	0.0021
	CCNA2	0.7143	0.2957 to 0.9028	0.0041
	CCNB2	0.6378	0.1620 to 0.8730	0.0141
	CCNE2	0.6373	0.1611 to 0.8728	0.0142
CHK2	CCNB1	0.7238	0.3136 to 0.9064	0.0034

In the table, on the left, is reported which kinase between WEE1, CHK1 and CHK2, has been correlated with the panel of DDR pathway representative genes. In the table it has been reported only Pearson values with a statistic significance $p > 0.01$.

Table S5. Primary sample's characteristics.

Sample ID	Subtype	Stage	% Blasts	CHK1			CHK2			WEE1		
				ΔCt CHK1	ΔΔCt CHK1/all	Fold Change CHK1/all	ΔCt CHK2	ΔΔCt CHK2/all	Fold Change CHK2/all	ΔCt WEE1	ΔΔCt WEE1/all	Fold Change WEE1/all
ALL#1	BCR−ABL1−positive	Diagnosis	80	0.535	1.603	0.329	3.665	0.741	0.598	−0.256	0.011	0.993
ALL#2	BCR−ABL1−positive	Relapse	70	−1.037	0.031	0.979	3.239	0.314	0.804	0.042	0.309	0.807
ALL#3	BCR−ABL1−positive	Relapse	80	0.045	1.113	0.462	5.915	2.990	0.126	1.809	2.076	0.237
ALL#4	BCR−ABL1−positive	Relapse	70	−1.046	0.022	0.985	3.365	0.440	0.737	−0.403	−0.136	1.099
ALL#5	BCR−ABL1−positive	Relapse	55	−1.938	−0.870	1.828	1.952	−0.972	1.962	−0.088	0.179	0.883
ALL#6	BCR−ABL1−negative	Relapse	80	−2.295	−1.227	2.342	0.831	−2.093	4.268	−2.355	−2.088	4.251
ALL#7	BCR−ABL1−negative	Diagnosis	70	−1.739	−0.671	1.592	1.505	−1.419	2.674	−0.617	−0.350	1.275

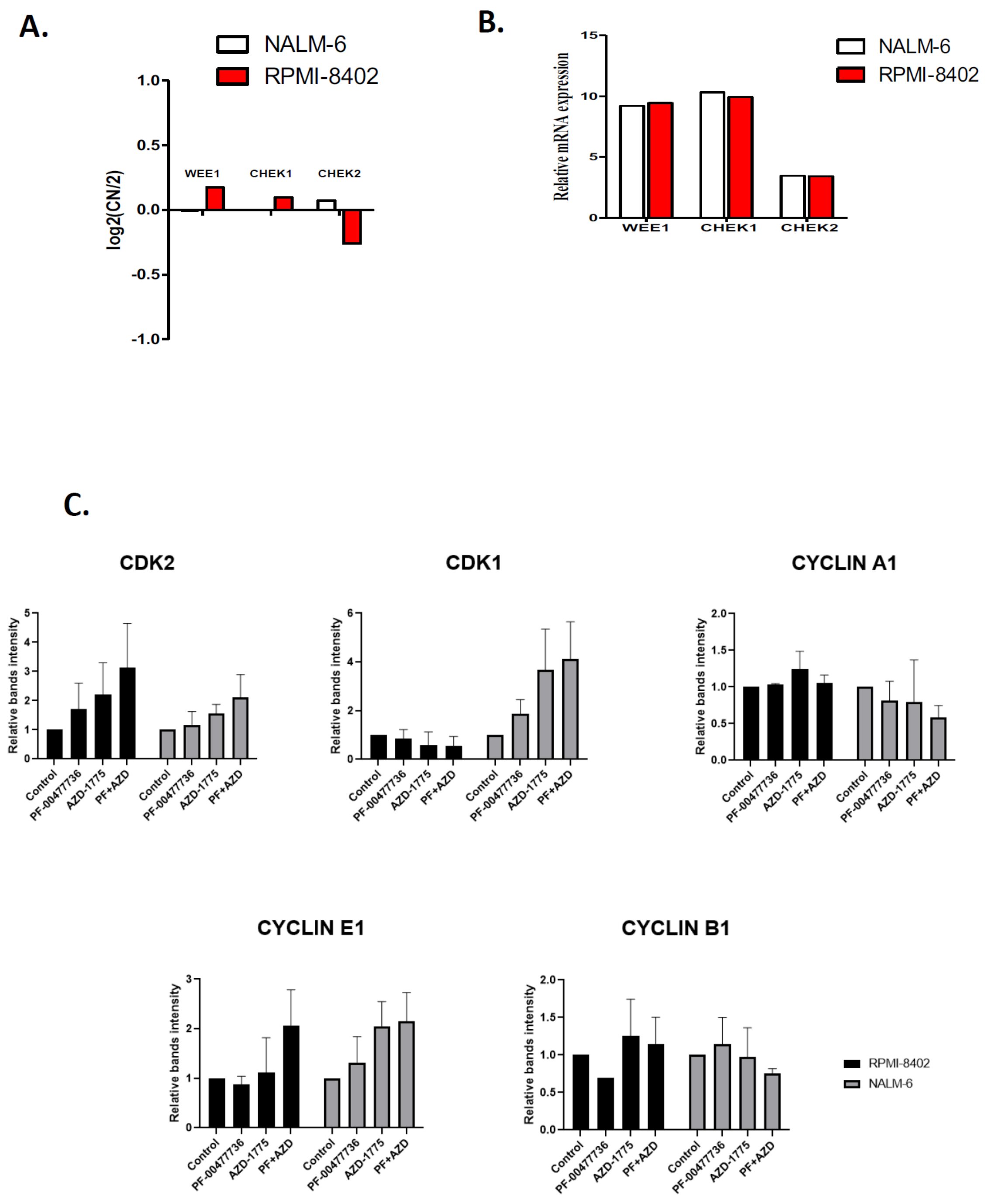

Figure S1. Copy number, mRNA and protein levels of genes involved in the response to the inhibitors. (**A**) Box-plots showing the relative copy number level of CHK1, CHK2 and WEE1 genes in NALM-6 and RPMI-8402 cell lines extracted from CCLE dataset. (**B**) Box-plots showing the gene expression level of Chek1, Chek2 and Wee1 genes in NALM-6 and RPMI-8402 cell lines extracted from CCLE dataset. (**C**)Histograms showing the relative protein expression of CDK1, CDK2, CYCLIN B1, CYCLIN E1 and CYCLIN A1 proteins in RPMI-8402 (Black) and NALM-6 (Gray) cells. Protein relative expression is reported as the average of at least two independent experiments.

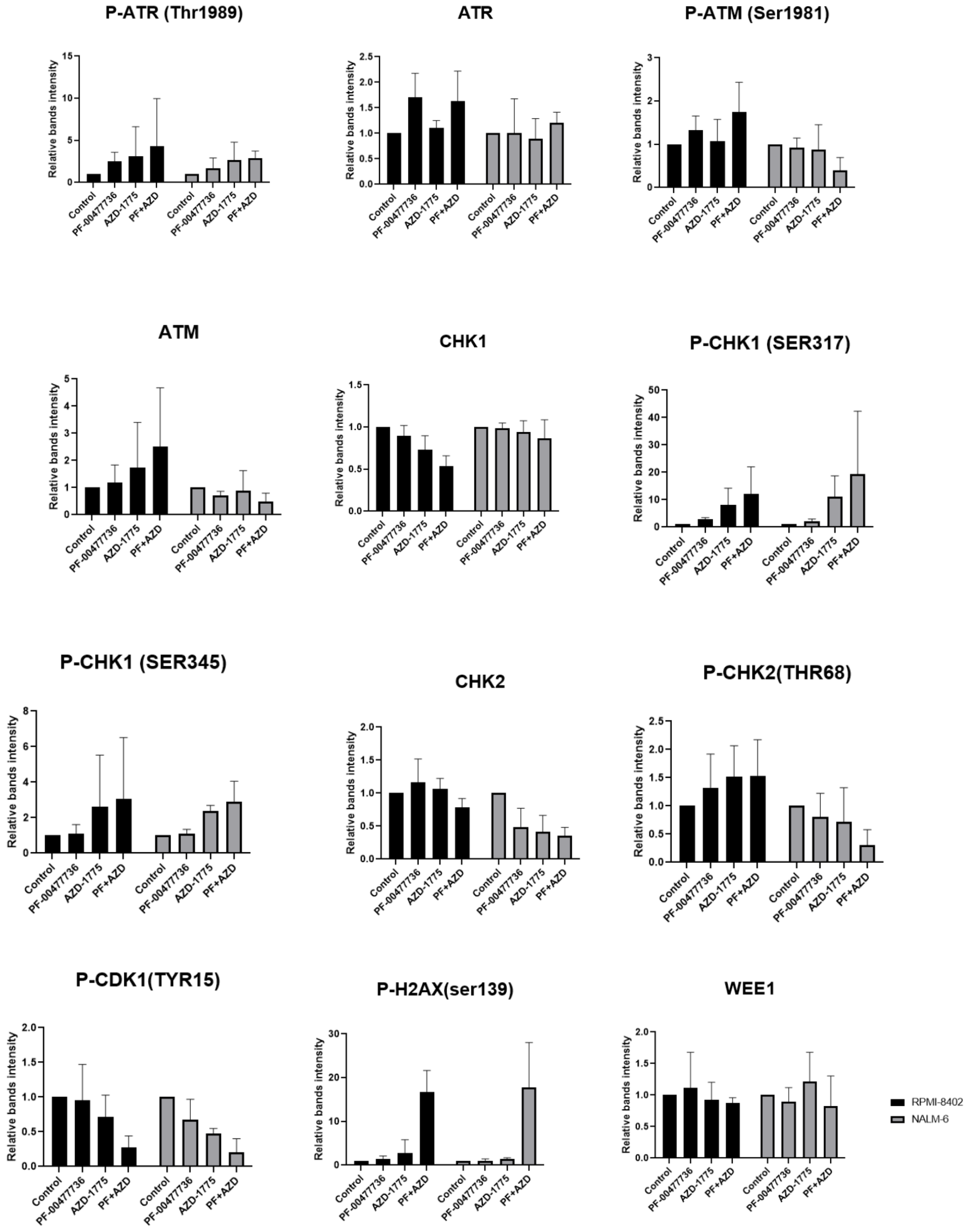

Figure S2. Histograms showing the relative protein expression of P-ATR(THR1989), ATR, P-ATM(SER1981), ATM, P-CHK1 (SER317), P-CHK1 (SER345), CHK1, P-CHK2 (THR68), CHK2, WEE1, P-CDK1(TYR15) and P-H2AX (SER139) proteins in RPMI-8402 (Black) and NALM-6 (Gray) cells. Protein relative expression is reported as the average of at least two independent experiments.

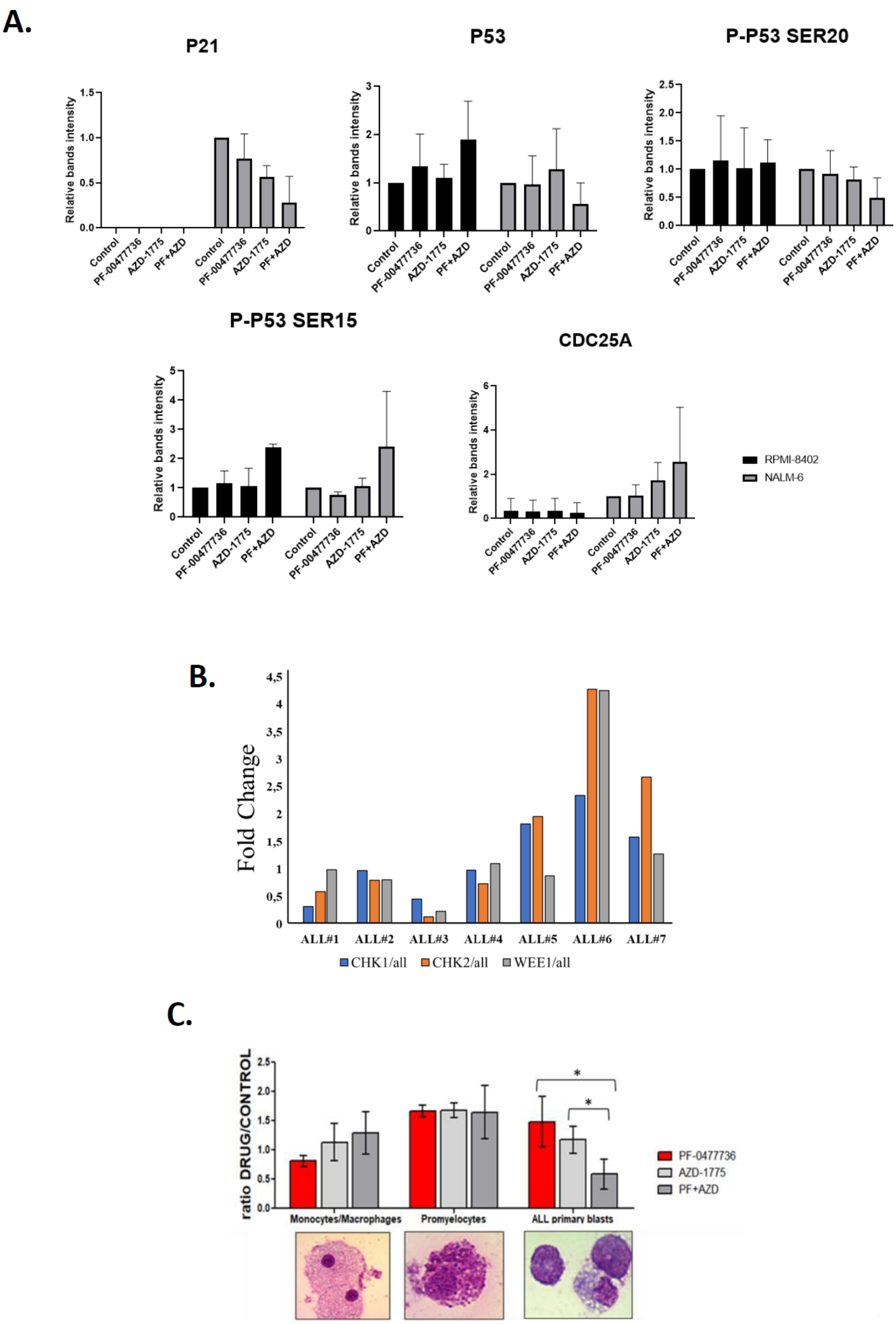

Figure S3. Effect of the combination in the protein level of ALL cell lines and relative mRNA level and morphological staining of primary B-ALL cells (**A**) Histograms showing the relative protein expression of P-P53(SER20), P-P53(SER15), P53, P21 and proteins in RPMI-8402 (Black) and NALM-6 (Gray) cells. Protein relative expression is reported as the average of at least two independent experiments (**B**) Histograms showing the normalized mRNA expression of Chek1, Chek2 and Wee1 mRNA of primary leukemic cells isolated from the BM of the seven B-ALL cases used for the clonogenic assays. The level of expression of each gene has been normalized with the average expression of the same gene in our cohort. (**C**) Morphological analysis of primary cells isolated from the colonies harvested in the clonogenic assays. The columns represent the ratio between the number of cells in the treated samples and in the controls (ratio Drug/Control). An average number of 300 cell/experimental condition was evaluated to quantify the number of cells. $p < 0.05$ one asterisk (*).

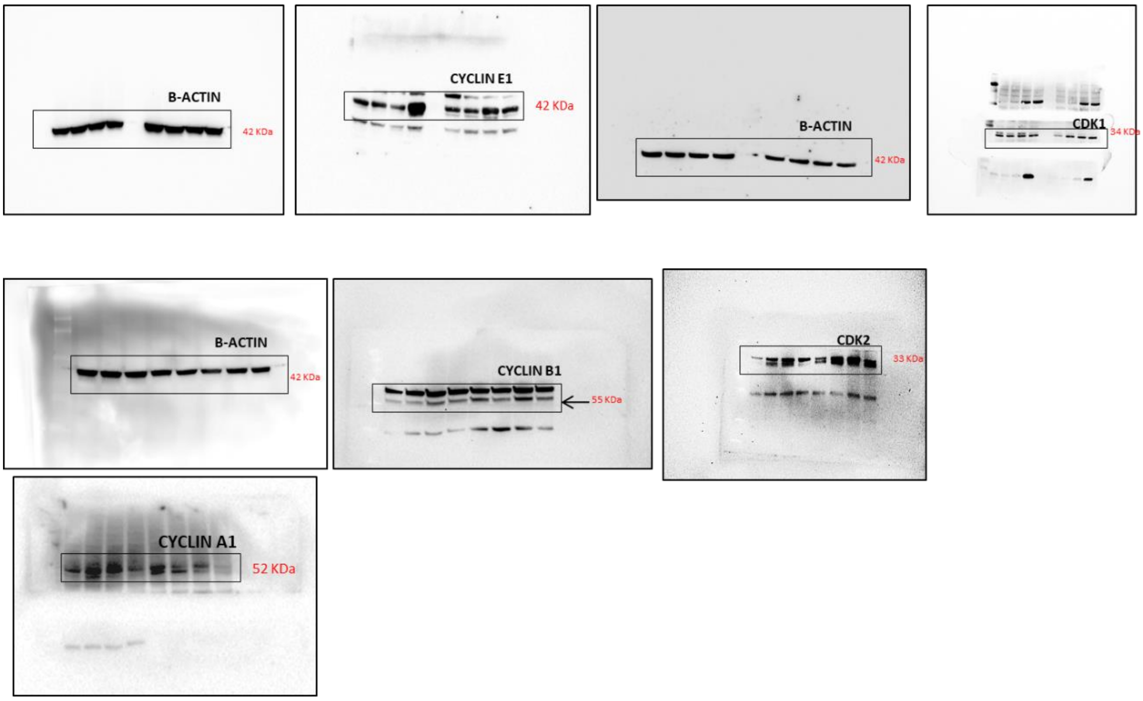

Figure S4. Whole western Blot immages.

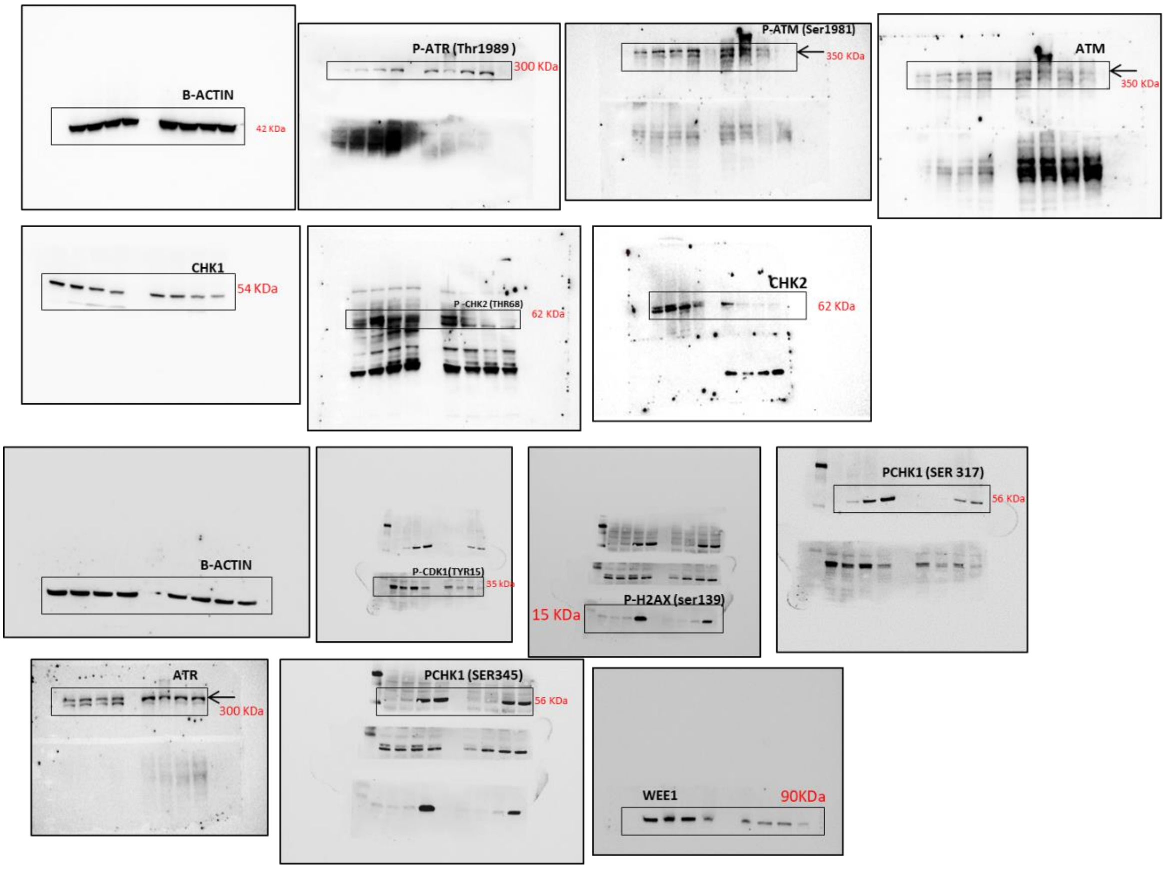

Figure S5. Whole western Blot immages.

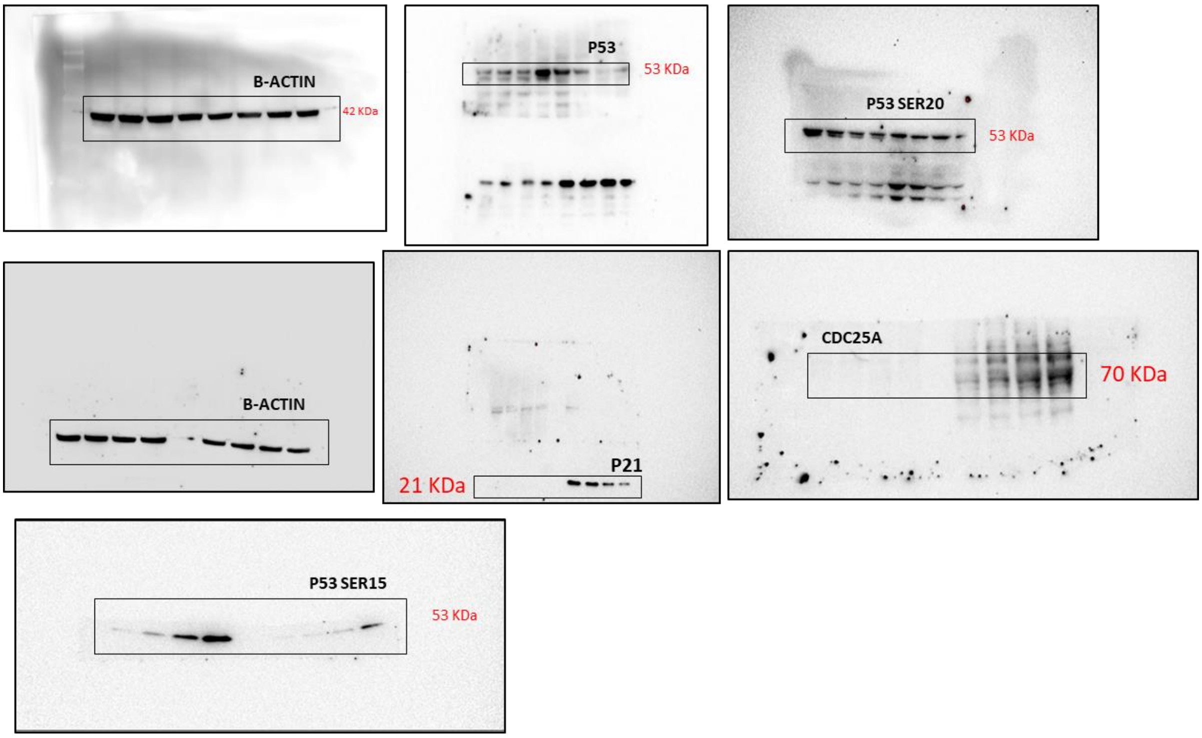

Figure S6. Whole western Blot immages.

© 2018 by the authors. Licensee MDPI, Basel, Switzerland. This article is an open access article distributed under the terms and conditions of the Creative Commons Attribution (CC BY) license (http://creativecommons.org/licenses/by/4.0/).
